# SUMO modifies GβL and mediates mTOR signaling

**DOI:** 10.1016/j.jbc.2024.105778

**Published:** 2024-02-21

**Authors:** Sophia Louise Lucille Park, Uri Nimrod Ramírez-Jarquín, Neelam Shahani, Oscar Rivera, Manish Sharma, Preksha Sandipkumar Joshi, Aayushi Hansalia, Sunayana Dagar, Francis P. McManus, Pierre Thibault, Srinivasa Subramaniam

**Affiliations:** 1 Department of Neuroscience, The Wertheim UF Scripps Institute, Jupiter, Florida, USA; 2 Institute for Research in Immunology and Cancer, Université de Montréal, Montréal, Quebec, Canada; 3 Department of Chemistry, Université de Montréal, Montréal, Quebec, Canada; 4 The Skaggs Graduate School of Chemical and Biological Sciences, The Scripps Research Institute, La Jolla, California, USA; 5 Norman Fixel Institute for Neurological Diseases, Gainesville, Florida, USA

**Keywords:** post-translational modification, SUMO interactive motif, protein–protein interaction, kinase signaling, SUMO mechanism, SUMO isoforms, lysine-site regulation, nutrient signaling, amino acid stimulation

## Abstract

The mechanistic target of rapamycin (mTOR) signaling is influenced by multiple regulatory proteins and post-translational modifications; however, underlying mechanisms remain unclear. Here, we report a novel role of small ubiquitin–like modifier (SUMO) in mTOR complex assembly and activity. By investigating the SUMOylation status of core mTOR components, we observed that the regulatory subunit, GβL (G protein β-subunit–like protein, also known as mLST8), is modified by SUMO1, 2, and 3 isoforms. Using mutagenesis and mass spectrometry, we identified that GβL is SUMOylated at lysine sites K86, K215, K245, K261, and K305. We found that SUMO depletion reduces mTOR–Raptor (regulatory protein associated with mTOR) and mTOR–Rictor (rapamycin-insensitive companion of mTOR) complex formation and diminishes nutrient-induced mTOR signaling. Reconstitution with WT GβL but not SUMOylation-defective KR mutant GβL promotes mTOR signaling in GβL-depleted cells. Taken together, we report for the very first time that SUMO modifies GβL, influences the assembly of mTOR protein complexes, and regulates mTOR activity.

The mechanistic target of rapamycin (mTOR) is a highly conserved serine/threonine kinase that senses amino acids (AAs), growth factors, and cellular energy levels to coordinate metabolism, cell growth, and survival (1). mTOR is the catalytic subunit of two distinct complexes: mTORC1, which is activated by nutrients and growth factors to regulate translation, cell growth, and metabolism, and mTORC2, which promotes cellular survival, proliferation, and cytoskeletal organization. While both mTOR and GβL (G protein β-subunit–like protein, also known as mLST8) are the core components that are assembled into both complexes, mTORC1 also includes Raptor (regulatory protein associated with mTOR), and mTORC2 contains Rictor (rapamycin-insensitive companion of mTOR). Together, these accessory proteins serve as scaffolds to dictate substrate specificity, recruit regulatory components to each complex, and govern kinase activity. However, it remains unclear how these regulators mechanistically facilitate the integration of multiple environmental cues to coordinate the dynamic assembly and activity of each complex based on cellular needs (1).

Reversible post-translational modifications influence multiple signaling pathways by altering protein–protein interactions, spatiotemporal dynamics, and signaling intensity (2, 3, 4). Given the complexity and dynamic nature of protein interactions that regulate mTOR signaling, several post-translational modifications regulate mTORC1 activity, complex assembly, and localization, including direct phosphorylation of Raptor (5) or mTOR on multiple sites (6) and K63-linked polyubiquitination of mTOR, RagA GTPase, and GβL (7, 8, 9, 10).

Like ubiquitin, SUMO (small ubiquitin–like modifier; three ubiquitously expressed paralogs in vertebrates: SUMO1, SUMO2, and SUMO3) is a conserved ∼10.5 kDa protein modification that is covalently attached to lysine residues on multiple substrate proteins in a dynamic and reversible manner (11). Although SUMOylation sites are usually within a ψKxE/D consensus motif, where ψ represents a bulky hydrophobic group, K the SUMOylated lysine, x any AA, and E/D a glutamic acid or an aspartic acid, a large number of substrates can be SUMOylated on nonconsensus lysine residues (12, 13, 14). SUMO conjugation is analogous to ubiquitination and requires activation with a single E1-activating enzyme (Uba2/Aos1 heterodimer), followed by transthiolation by the sole E2-conjugating enzyme, Ubc9, and attachment to target substrates by Ubc9 alone or in conjunction with a SUMO-E3 ligase. We have also previously demonstrated that E1 and E2 enzymes can be reciprocally SUMOylated by each other, a process termed “cross-SUMOylation” (15). Finally, SUMO-modified proteins are deconjugated by paralog-sensitive SUMO proteases (SENPs). SUMOylation of target substrates is well documented to establish a diverse range of cellular processes, including gene expression, intracellular trafficking, protein degradation, and meiotic recombination (3, 16, 17, 18).

Intriguingly, SUMO has been shown to regulate multiple kinases (19, 20) as well as the activity and intracellular localization of PTEN (21, 22), Akt (23, 24), AMPK (25, 26), and TBK1 (27). In addition, the SUMO-specific isopeptidase SENP3 is directly phosphorylated by mTOR (28), confirming that mTOR interacts with SUMO-conjugating machinery. SUMO1 and SUMO3 are dispensable in normal development, but SUMO2 deletion is embryonically lethal (29, 30, 31). Furthermore, *Sumo1* KO mice have reduced body weight and are resistant to diet-induced obesity (29, 32), similarly to adipose-specific *Raptor* KO mice (33). We have previously demonstrated that the striatal-enriched small G protein Rhes that harbors N-terminal SUMO-E3-like domain that SUMOylates mutant huntingtin (mHTT) and directly binds and activates mTOR to promote Huntington's disease pathogenesis (15, 34, 35, 36, 37). Thus, we hypothesized that SUMOylation may regulate mTOR signaling and investigated it by employing biochemical and proteomic approaches.

## Results

### GβL, the constitutively bound regulatory subunit of mTOR, is modified by SUMO

**Figure 1:**
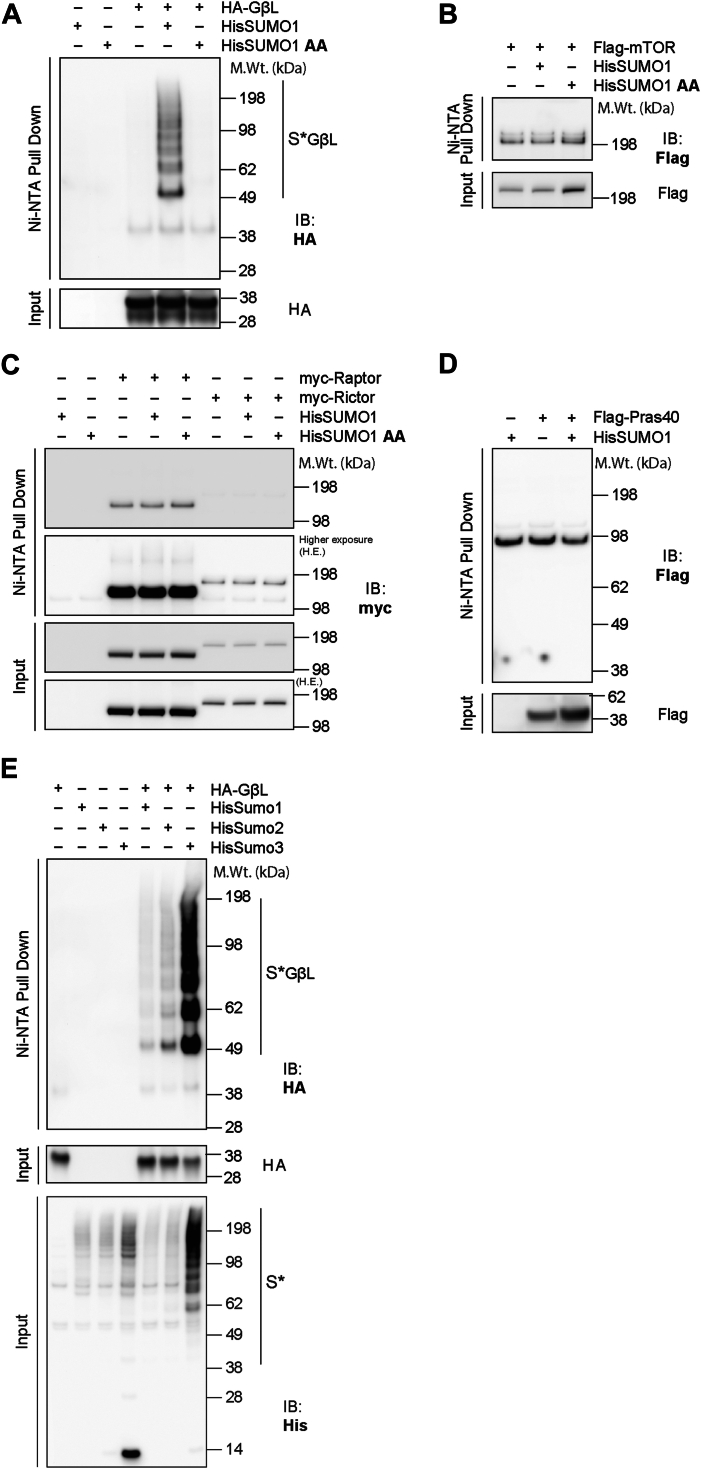
**GβL is the primary mTOR complex component that is SUMOylated.***A*, representative Western blot of Ni–NTA enrichment of proteins in denaturing conditions and corresponding input from HEK293 cells transfected with His-SUMO1 and HA-GβL in full media conditions, showing an enrichment of HA-GβL high molecular weight conjugates with His-SUMO1 but not His-SUMO1 amino acid (AA) (conjugation-defective mutant). *B*–*D*, Ni–NTA enrichment of His-SUMO1 conjugates associated with FLAG-mTOR (*B*), myc-Raptor or myc-Rictor (*C*), or FLAG-Pras40 (*D*) as in *A*. *E*, Ni–NTA enrichment of His-SUMO1, 2, and 3 conjugates associated with HA-GβL (indicated by S∗ GβL) and corresponding inputs as in *A*. GβL, G protein β-subunit–like protein; HEK293, human embryonic kidney 293 cell line; mTOR, mechanistic target of rapamycin; Ni, nickel; NTA, nitrilotriacetic acid; Raptor, regulatory protein associated with mTOR; Rictor, rapamycin-insensitive companion of mTOR; SUMO, small ubiquitin–like modifier.

To understand the role of SUMOylation on mTOR activity, we first tested whether any mTOR components can be SUMO modified. To do so, we transiently overexpressed HA-GβL, myc-Rictor, myc-Raptor, FLAG-PRAS40, and FLAG-mTOR in the presence or the absence of His-SUMO1 in human embryonic kidney 293 (HEK293) cells, followed by nickel–nitrilotriacetic acid (Ni–NTA) denaturing enrichment (38). Since SUMOylated proteins exist at low stoichiometry and SUMO proteases rapidly remove SUMOylation in native lysates (39), this strategy helped to prevent cleavage of SUMO from corresponding target substrates and allowed for further enrichment of potential SUMO substrates. As shown in Figure 1, high molecular weight conjugates of GβL are enriched only in the presence of His-SUMO1 (S∗ GβL, Fig. 1*A*), starting with a primary band at ∼49 kDa that is consistent with the ∼11 kDa SUMO1 modification size. SUMO1 lacking the essential diglycine conjugation motif (HisSUMO1-AA), which cannot be covalently attached to target lysines (40), failed to enrich high molecular weight species of GβL (Fig. 1*A*). This result suggests that SUMO modification on GβL is specific and requires the covalent attachment of SUMO1 to GβL. Preliminary data also indicated that GβL is heavily modified by SUMO1 compared with other mTOR components (mTOR, Raptor, Rictor, or PRAS40; Fig. 1, *B*–*D*). Furthermore, high molecular weight conjugates of GβL are enriched in the presence of His-SUMO1, His-SUMO2, and His-SUMO3, suggesting all SUMO isoforms can modify GβL (Fig. 1*E*). Together, these biochemical observations reveal that GβL, a primary core component of mTOR, is strongly modified by SUMO1, SUMO2, and SUMO3.

### GβL is SUMO modified at K86, K215, K245, K261, and K305

**Figure 2:**
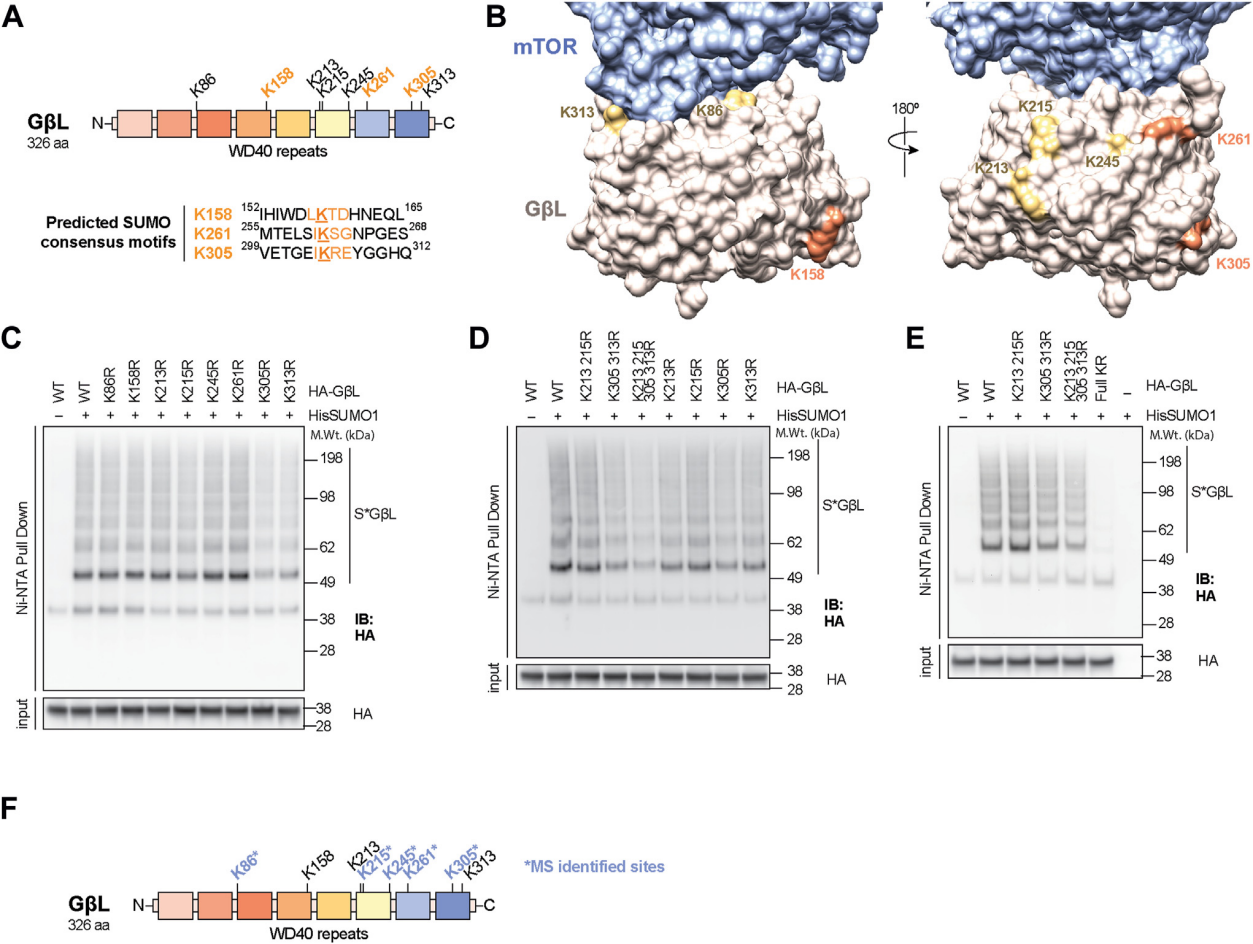
**GβL is SUMOylated at multiple lysines.***A*, primary sequence domain structure of GβL with predicated SUMO-modification sites for KR mutagenesis. *B*, 3D representation of the mTOR–GβL interface demonstrating that all lysines on GβL are surface exposed and accessible. SUMO consensus sites are shown in *orange*, nonconsensus sites in *yellow*, mTOR in *blue*, and GβL in *tan*. The interface was modeled using the reconstructed density from Protein Data Bank (ID: 5FLC). *C*, Western blot analysis of Ni–NTA denaturing pull down and corresponding input from HEK293 cells transfected with His-SUMO1 and WT or single KR mutant HA-GβL plasmids. *D* and *E*, Western blot analysis as in *C* from HEK293 cells transfected with HA-GβL KR mutant plasmids as indicated. *F*, SUMO-conjugated lysine identification on HA-GβL by LC–MS/MS (depicted with ∗) and corresponding location in the domain structure. GβL, G protein β-subunit–like protein; HEK293, human embryonic kidney 293 cell line; MS, mass spectrometry; mTOR, mechanistic target of rapamycin; Ni, nickel; NTA, nitrilotriacetic acid; SUMO, small ubiquitin–like modifier.

To identify the potential lysine residue(s) on GβL that are modified by SUMO1, we performed lysine to arginine (K to R) site-directed mutagenesis. GβL contains eight surface-exposed lysine residues distributed across the primary sequence (K86, K158, K213, K215, K245, K261, K305, and K313; Fig. 2, *A* and *B*) (41), with three predicted SUMO consensus motifs (K158, K261, and K305). To identify potential lysine modification sites, we generated single, double, quadruple, and full KR mutants of GβL. First, we transfected HEK293 cells with WT HA-GβL or different KR HA-GβL mutant constructs together with His-SUMO1, followed by enrichment of SUMO conjugates using Ni–NTA pulldown. If one or more of these lysines are conjugated by SUMO1, then arginine substitution should prevent conjugation, and Ni–NTA enrichment of the corresponding construct should be impaired compared with WT GβL. Mutagenesis of single lysines showed a varied degree of deficit in GβL SUMOylation (Fig. 2*C*). The deletion of SUMO consensus sites (K158, K261, and K305) did not abrogate GβL SUMOylation, indicating that either (1) SUMOylation shifted to nonconsensus sites in the mutants or (2) GβL SUMOylation can occur on nonconsensus sites. Double and quadruple lysine mutation sites revealed that the predominant SUMO1 acceptor sites on GβL are K213, K215, K305, and K313 (Fig. 2*D*), indicated by the reduced accumulation of high molecular weight conjugates when these sites were mutated. Mutation of all lysine sites (HA-GβL full KR) eliminated the SUMO modification of GβL (Fig. 2*E*), indicating that SUMOylation of GβL is dynamic and fluid in nature. To better elucidate potential SUMO modification sites on GβL, we performed mass spectrometry (MS) on Ni–NTA pulldowns of HEK293 cells transfected with HA-GβL and His-mSUMO3 (compatible for MS detection (42, 43, 44)) and confirmed that GβL is SUMO3 modified at residues K86, K215, K245, K261, and K305 (Fig. 2*F* and Data S1). Our previous study also identified that GβL is SUMOylated in HEK293 cells stably expressing SUMO3 (43). Furthermore, endogenous GβL was also shown to be SUMO modified at K305 by MS analysis (45). Taken together, these results from site-directed mutagenesis and MS analysis demonstrat that GβL is SUMOylated at multiple lysines, which may in turn regulate mTOR signaling *via* modulating protein–protein interactions.

### Recombinant GβL cannot be SUMOylated *in vitro*

**Figure 3:**
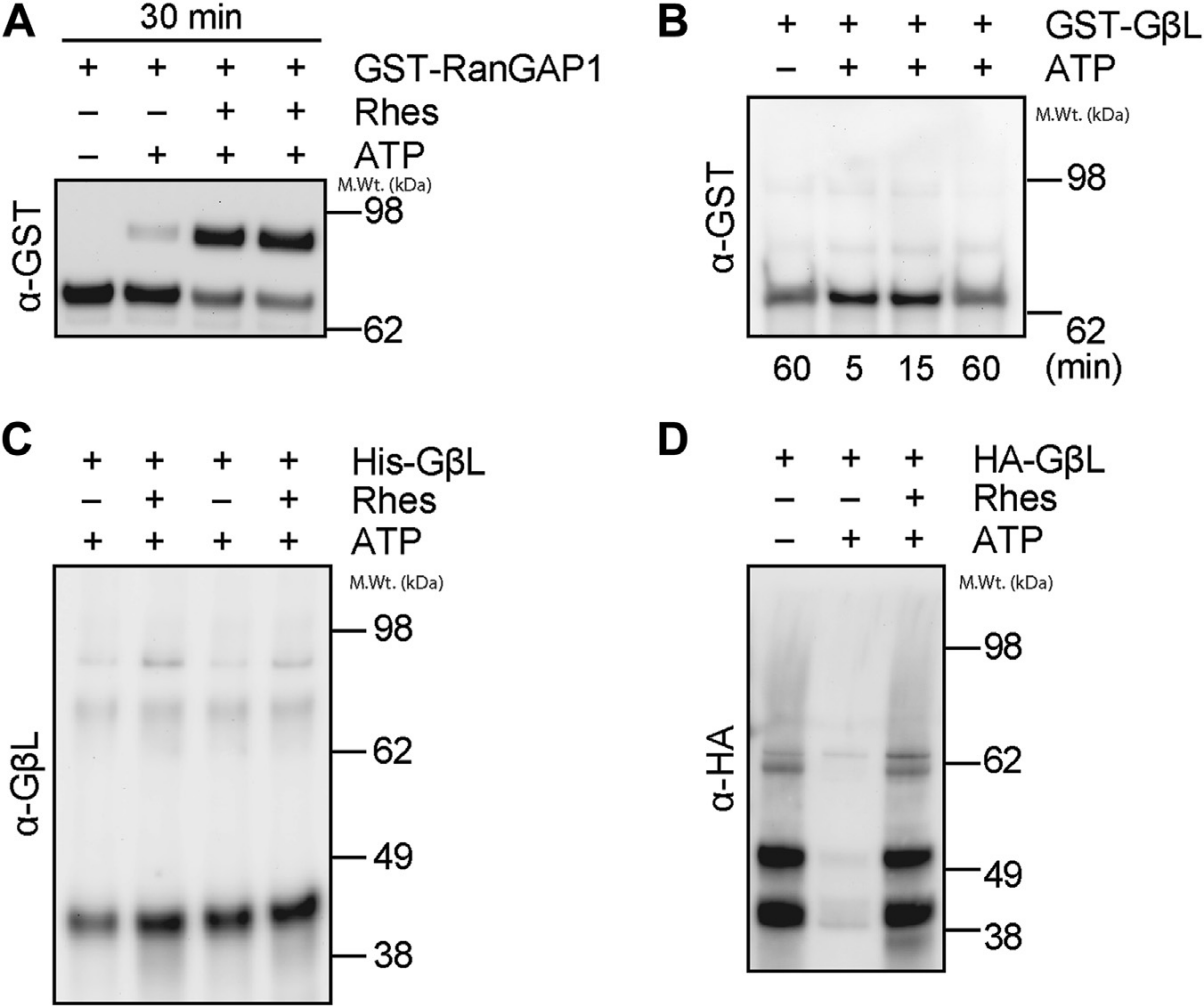
***In vitro* SUMOylation of GβL. *In vitro* SUMOylation** reactions were performed with RanGAP1 or GβL, E1, and E2 in the presence and absence of ATP (5 mM) or Rhes (200 ng) as indicated. *A* and *B*, *in vitro* SUMOylation of recombinant GST-RanGAP1 (*A*) or GST-GβL (*B*), detected using anti-GST-HRP. *C*, *in vitro* SUMOylation of purified recombinant His-GβL, detected using anti-GβL. *D*, *in vitro* SUMOylation on beads from HA immunoprecipitates from HEK293 cells transfected with HA-GβL, detected using anti-HA. GβL, G protein β-subunit–like protein; GST, glutathione-S-transferase; HEK293, human embryonic kidney 293 cell line; HRP, horseradish peroxidase; SUMO, small ubiquitin–like modifier.

SUMOylation of target substrates requires specific conjugation machinery to occur *in vivo*, and E3-SUMO ligases facilitate substrate specificity and enhance SUMO transfer from the E2-SUMO-conjugating enzyme, Ubc9. We tested whether GβL could be SUMOylated using purified recombinant E1 and E2 *in vitro*. As shown before, glutathione-*S*-transferase (GST)-RanGAP1 is rapidly SUMOylated in the presence of E1 and E2, and this effect, as expected, is enhanced in the presence of the E3 ligase, Rhes (15, 34) (Fig. 3*A*). In contrast, when purified recombinant GST-GβL or His-GβL were incubated in the presence of E1 and E2 and Rhes, we did not observe any high molecular weight conjugates indicative of SUMOylation of GβL (Fig. 3, *B* and *C*). Furthermore, immunoprecipitation (IP) of HA-GβL from HEK293 cells and subsequent *in vitro* SUMOylation revealed that GβL was not SUMOylated in the presence of E1 and E2 or in the presence of the E3 ligase, Rhes (Fig. 3*D*). These data suggest that SUMO E1 and E2 are not sufficient to SUMOylate GβL *in vitro*, indicating that an E3 ligase other than Rhes is required for the SUMOylation of GβL.

### SUMO regulates AA-induced activation of mTORC1 signaling

**Figure 4:**
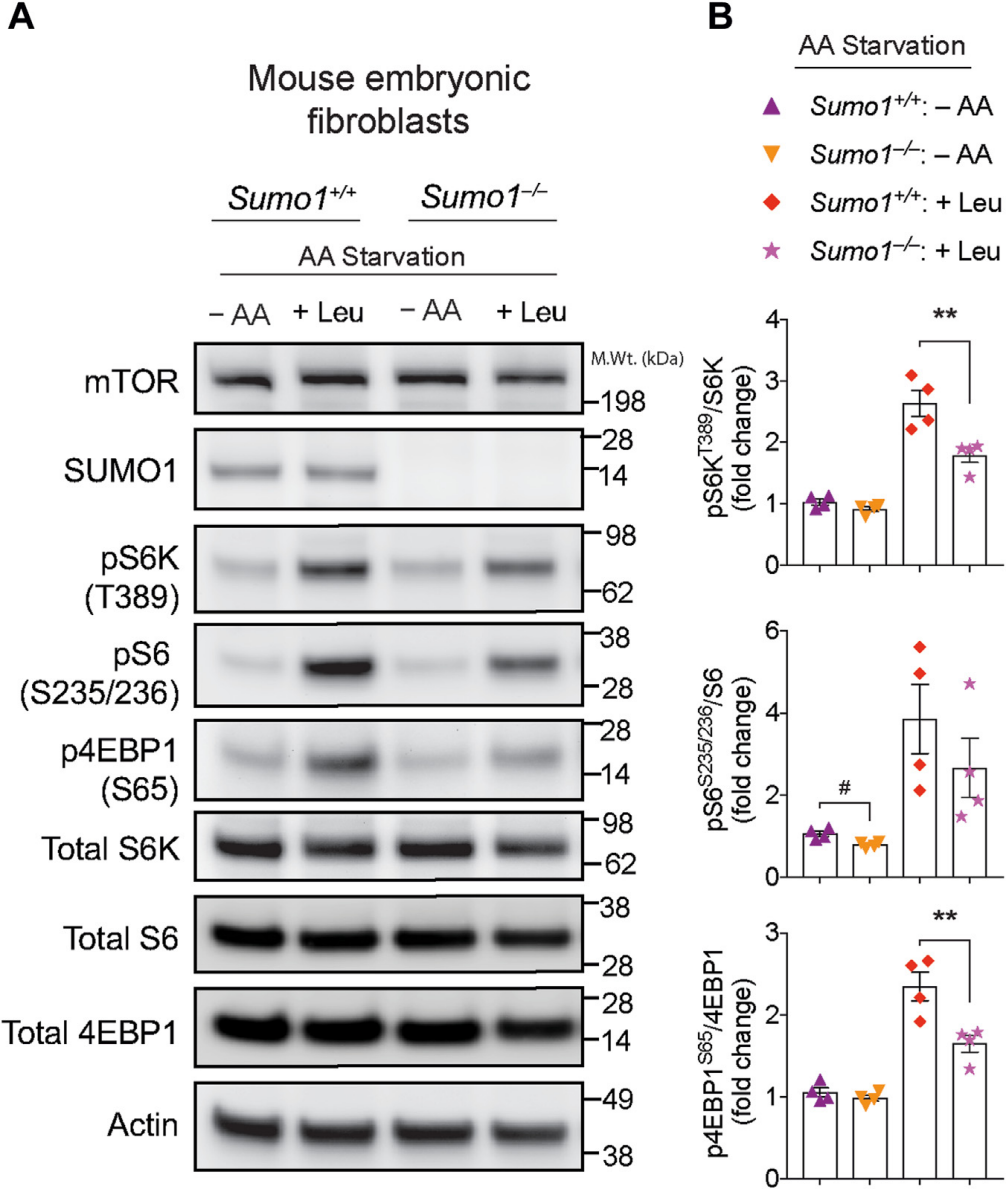
**mTORC1 activity is altered in *SUMO1***^***−/−***^**MEFs.***A*, representative Western blot showing indicated phosphorylation of mTORC1 substrates in WT (*Sumo1*^*+/+*^) and Sumo1 KO (*Sumo1*^*−/−*^) primary MEFs grown in F12 media starved of amino acids (−AA) and stimulated with 3 mM l-leucine (+Leu). *B*, quantification of indicated proteins from *A*. Error bars represent mean ± SEM, ∗∗*p* < 0.01 by one-way ANOVA/Tukey’s multiple comparison test, #*p* < 0.05 by Student’s *t* test. MEF, mouse embryonic fibroblast; mTOR, mechanistic target of rapamycin.

Since we found that the mTOR regulatory subunit GβL is SUMOylated, we then examined if the loss of *Sumo1* influences mTORC1 activity. To test this hypothesis, we generated *Sumo1* WT (*Sumo1*^*+/+*^) and *Sumo1* KO (*Sumo1*^*−/−*^) primary mouse embryonic fibroblasts (MEFs) at E13.5 and measured mTORC1 activity (pS6K^T389^, pS6^S235/236^, and p4EBP1^S65^) under conditions of essential AA starvation (−AA) in the presence or absence of leucine stimulation (+Leu). We observed a slight decrease in the phosphorylation of the S6K target, pS6^S235/236^, in AA-starved *Sumo1*^*−/−*^ MEFs, compared with *Sumo1*^*+/+*^ MEFs (Fig. 4, *A* and *B*). Upon leucine stimulation, we found a strong reduction in the phosphorylation of direct mTOR targets, pS6K^T389^ and p4EBP1^S65^, in *Sumo1*^*−/−*^ MEFs compared with *Sumo1*^*+/+*^ (Fig. 4, *A* and *B*). We also observed a noticeable decrease in pS6^S235/236^ upon leucine stimulation (Fig. 4, *A* and *B*). Furthermore, insulin-induced phosphorylation of the mTORC2 target (pAkt 473) or PI3K target (pAkt 308) was unaltered in *Sumo1*^*−/−*^ MEFs, compared with *Sumo1*^*+/+*^ (Fig. S1). These results indicate that loss of *Sumo1* impairs mTORC1 activity, but not mTORC2 or PI3K mediated Akt phosphorylation.

**Figure 5:**
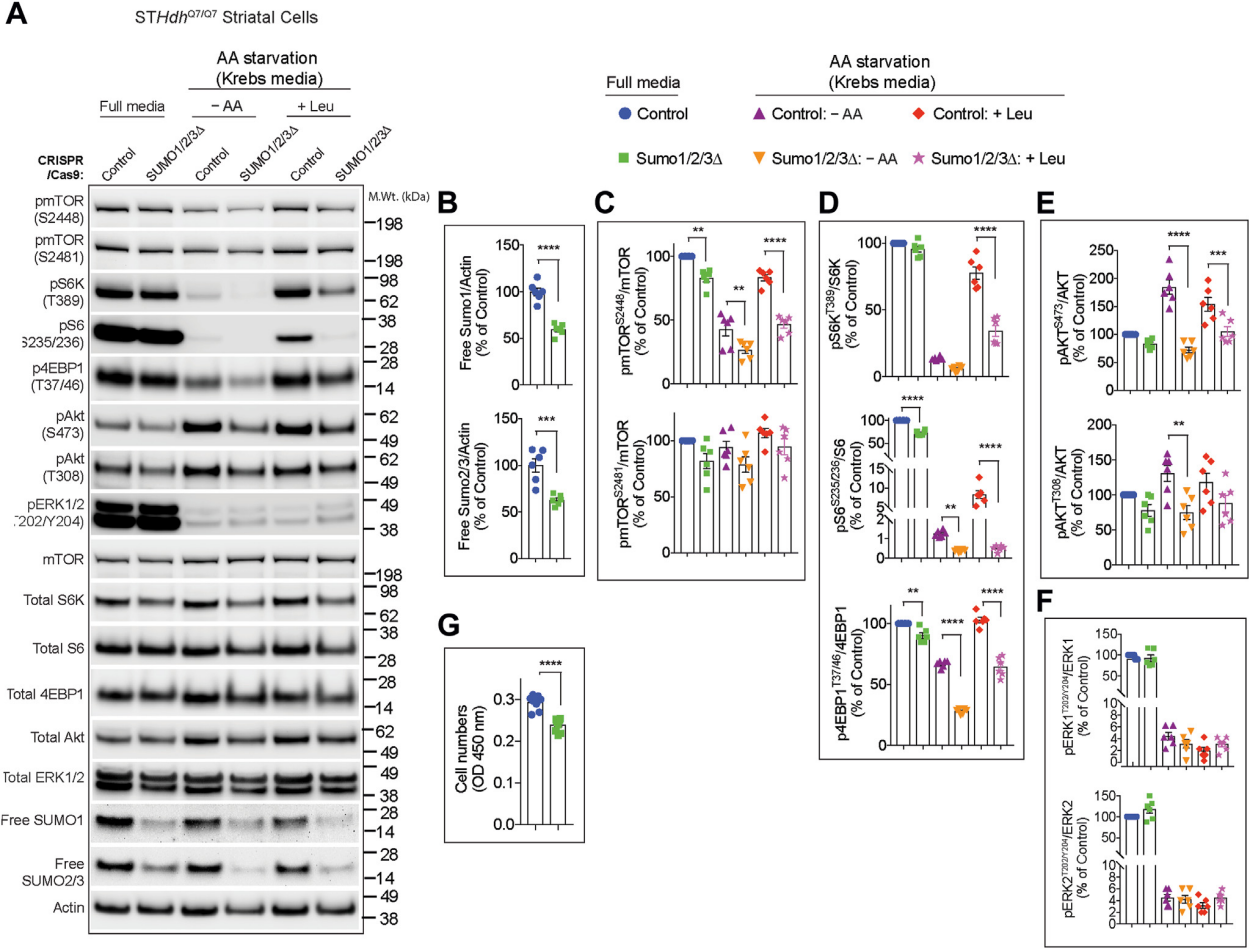
**mTOR activity in SUMO-depleted striatal neuronal cells.***A*, representative Western blot showing indicated signaling proteins in striatal control CRISPR- (control) or SUMO1/2/3-depleted (SUMO1/2/3Δ) cells in full media, deprived of amino acids (AAs) in Krebs buffer (−AA), and starved and stimulated with 3 mM leucine (+Leu). *B*–*F*, quantification of indicated proteins from *A*. *G*, cell proliferation assay using cell counting kit-8 (CCK-8) assay. Error bar represents mean ± SEM, ∗∗*p* < 0.01, ∗∗∗*p* < 0.001, ∗∗∗∗*p* < 0.0001 by one-way ANOVA/Tukey’s multiple comparison test. mTOR, mechanistic target of rapamycin; SUMO, small ubiquitin–like modifier.

Because previous studies have shown that SUMO2 and SUMO3 can compensate for SUMO1 loss of function in mice (29, 30), we hypothesized that SUMO2 or SUMO3 might compensate for SUMO1 and/or in addition contribute in regulating mTORC1 activity. To experimentally test this hypothesis, we employed SUMO1/2/3-depleted striatal neuronal cells (ST*Hdh*^*Q7*^*/*^*Q7*^) generated using CRISPR–Cas-9 technology (SUMO1/2/3Δ cells), which displayed ∼40% reduction in unconjugated SUMO1 and SUMO2/3 levels compared with control cells (46) (Fig. 5, *A* and *B*). We then compared mTORC1 signaling between control and SUMO1/2/3Δ cells in full media conditions (serum + AAs), AA starvation (Krebs media), and starvation followed by leucine stimulation (+Leu). We found a significant decrease in the phosphorylation of mTOR at Ser^2448^ (a target of PI3K (47)) in SUMO1/2/3Δ cells compared with control cells in all conditions (Fig. 5, *A* and *C*). However, the phosphorylation of mTOR at Ser^2481^, an autophosphorylation site (48), is not significantly affected in full media or by starvation or leucine stimulation in SUMO1/2/3Δ cells (Fig. 5, *A* and *C*). In full media and AA starvation conditions, the phosphorylation of both the mTORC1 target (p4EBP1^T37/46^) and S6K target (pS6^S235/236^) were significantly attenuated in SUMO1/2/3Δ cells, compared with control cells (Fig. 5, *A* and *D*). Upon leucine stimulation, while control cells showed a strong activation of mTORC1 signaling (pS6K^T389^, pS6^S235/236^, and p4EBP1^T37/46^), this was markedly decreased in SUMO1/2/3Δ cells (Fig. 5, *A* and *D*). These results indicate that depletion of all three SUMO isoforms robustly decreases AA-induced activation of mTORC1 in striatal neuron cells.

Next, we investigated whether SUMOylation affects the activation of mTORC2 (pAkt^S473^) or PI3K (pAkt^T308^) signaling. In full media conditions, the levels of pAkt^S473^ or pAkt^T308^ are comparable between control and SUMO1/2/3Δ cells (Fig. 5, *A* and *E*). Upon starvation, consistent with previous reports (49, 50, 51), we found that the pAkt^S473^ and pAkt^T308^ levels are increased in control cells, but not in SUMO1/2/3Δ cells (Fig. 5, *A* and *E*). Leucine addition had no further impact on pAkt^S473^ and pAkt^T308^ levels in control cells or SUMO1/2/3Δ cells (Fig. 5, *A* and *E*). These observations indicate that SUMOylation regulates the starvation-induced upregulation of Akt activity mediated by mTORC2 and PI3K. In contrast, we did not observe any changes in the levels of pERK^T202/Y204^ in SUMO1/2/3Δ cells compared with control cells (Fig. 5, *A* and *F*). As expected, pERK^T202/Y204^ is downregulated upon starvation (52), which was similar and unaffected upon Leu addition in both SUMO1/2/3Δ and control cells (Fig. 5, *A* and *F*). Moreover, insulin-induced mTORC2 (pAkt^473^) and PI3K (pAkt^308^) was similar between control and SUMO1/2/3Δ cells (Fig. S2). These results indicate that SUMO selectively regulates the specific starvation- and AA-mediated phosphorylation status of mTOR signaling without significantly interfering with ERK signaling. Finally, we tested whether diminished mTORC1 signaling in SUMO1/2/3Δ cells affects cell viability or proliferation. We did not observe floating cells indicative of cell death, but we found a decreased cell number in SUMO1/2/3Δ cells compared with control cells (Fig. 5*G*). As mTOR signaling is implicated in cell growth and proliferation (53), we conclude that diminished proliferation of SUMO1/2/3Δ cells may be due to reduced mTOR signaling.

### SUMO regulates the mTORC1 and mTORC2 protein complex formation

**Figure 6:**
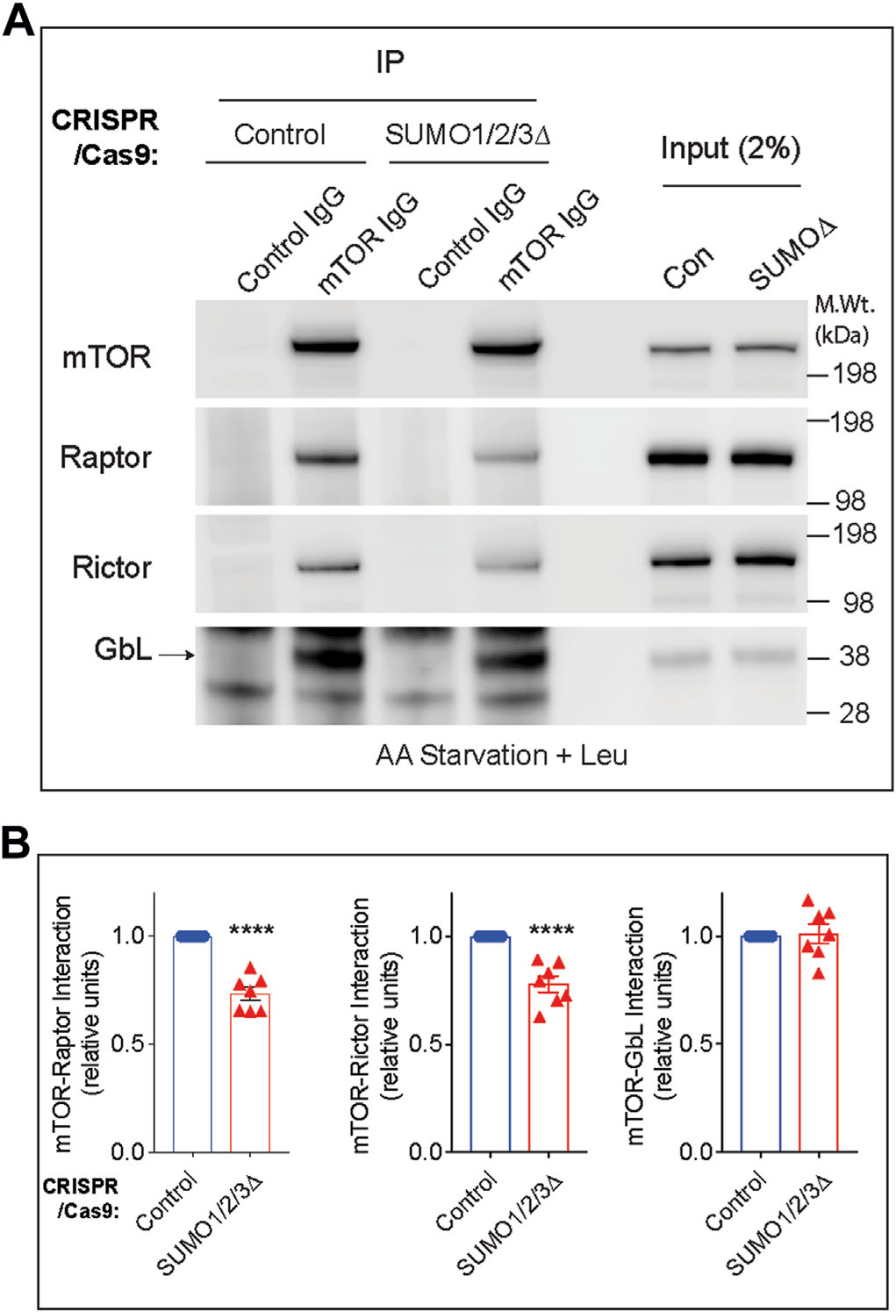
**SUMO influences mTOR complex formation in striatal neuronal cells.***A*, immunoprecipitation of mTOR with mTOR IgG or control IgG and Western blotting for endogenous Raptor, Rictor, or GβL from striatal control CRISPR- or SUMO1/2/3-depleted (SUMO1/2/3Δ) cells in AA-starved (Krebs) and stimulated with 3 mM leucine (+Leu). *B*, quantification of indicated protein interactions from *A*. Error bar represents mean ± SEM, ∗∗∗∗*p* < 0.001 by Student’s *t* test. AA, amino acid; GβL, G protein β-subunit–like protein; IgG, immunoglobulin G; mTOR, mechanistic target of rapamycin; Raptor, regulatory protein associated with mTOR; Rictor, rapamycin-insensitive companion of mTOR; SUMO, small ubiquitin–like modifier.

We then wondered how SUMO might mechanistically regulate mTOR signaling. One possibility is that SUMO regulates complex formation of mTORC1 and mTORC2. To address this, we carried out IP experiments against mTOR and assessed its interaction with Raptor (mTORC1), Rictor (mTORC2), and GβL (a constitutively bound component of both mTORC1 and mTORC2) in control and SUMO1/2/3Δ cells. As expected, mTOR immunoglobulin G (IgG), but not control IgG, readily immunoprecipitated mTOR and coprecipitated Raptor, Rictor, and GβL (Fig. 6, *A* and *B*). However, the abundance of mTOR–Raptor and mTOR–Rictor interactions were diminished in SUMO1/2/3Δ cells compared with control cells, whereas the interaction with mTOR–GβL was unaffected (Fig. 6, *A* and *B*). Note, the ∼30% decrease in the interaction is highly significant (Fig. 6, *A* and *B*) as SUMO1/2/3Δ cells show only ∼40% depletion of SUMO, compared with control cells (Fig. 5*B*), indicating that SUMO selectively and strongly regulates the interaction of mTOR with Raptor and Rictor.

### SUMOylation-defective mutant of GβL fails to activate mTOR signaling

**Figure 7:**
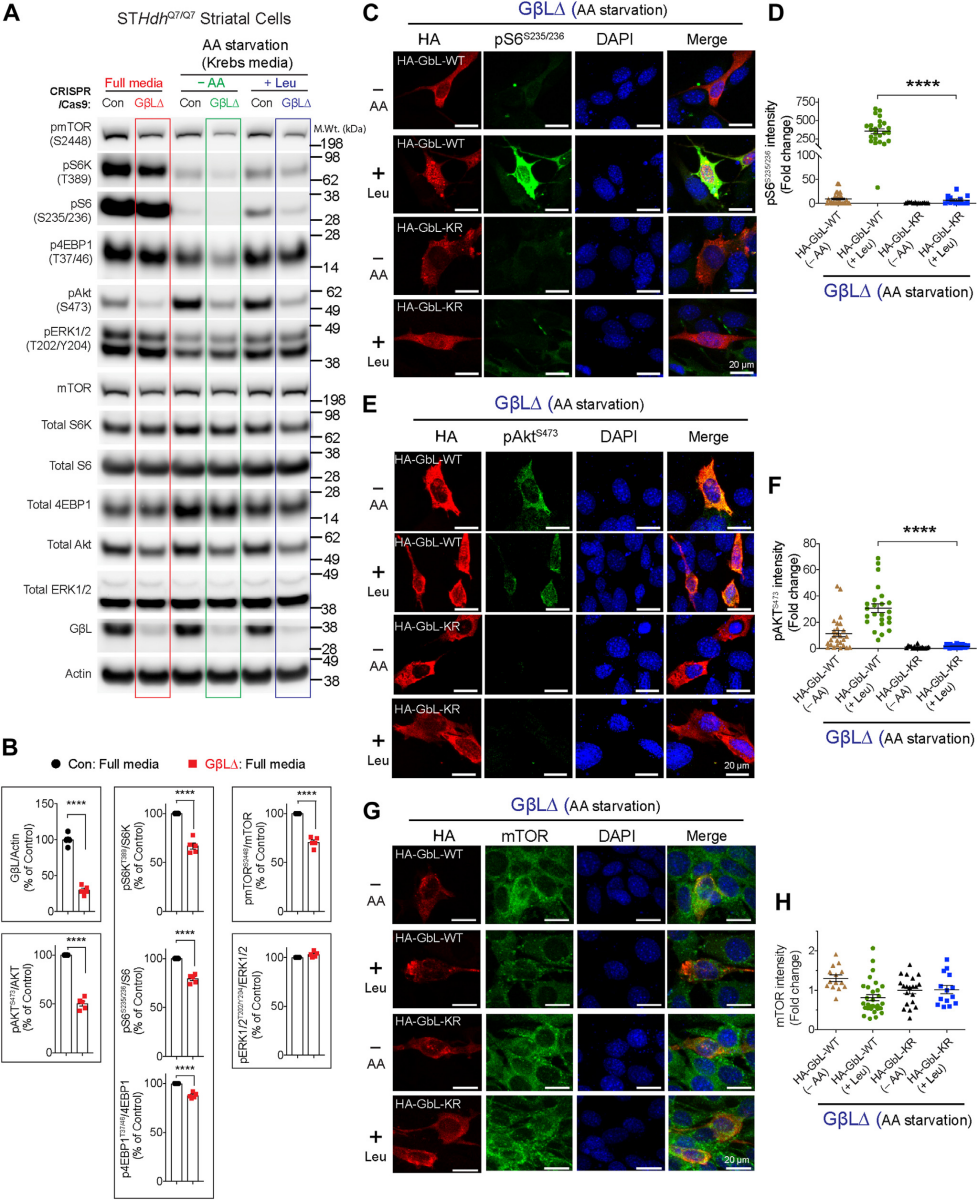
**Effect of SUMOylation-defective GβL on mTOR signaling.***A*, representative Western blot showing indicated signaling proteins in striatal control CRISPR- (control) or GβL-depleted (GβLΔ) cells in full media, deprived of amino acids (AAs) in Krebs buffer (−AA), and starved and stimulated with 3 mM leucine (+Leu). *B*, quantification of indicated proteins in full media from *A*. Error bar represents mean ± SEM, ∗∗∗∗*p* < 0.0001 by Student’s *t* test. *C*–*H*, representative confocal immunofluorescence images and quantification (n = 13–30) of the signal intensity of pS6^S235/236^ (*C* and *D*), pAkt^S473^ (*E* and *F*), and mTOR (*G* and *H*) in GβLΔ cells expressing HA-GβL-WT or HA-GβL-KR mutant in AA-starved (Krebs, –AA) or starved and stimulated with 3 mM leucine (+Leu). DAPI was used for nuclear stain. Error bar represents mean ± SEM, ∗∗∗∗*p* < 0.001 by one-way ANOVA/Tukey’s multiple comparison test. DAPI, 4′,6-diamidino-2-phenylindole; GβL, G protein β-subunit–like protein; mTOR, mechanistic target of rapamycin; SUMO, small ubiquitin–like modifier.

Because we found that the mTOR and GβL interaction was unaffected in SUMO-depleted cells (Fig. 6*A*), we hypothesized that SUMOylation of GβL may participate in the regulation of mTOR activity. To test this, we generated GβL-depleted (GβLΔ) striatal neuronal cells using CRISPR–Cas9 tools. We obtained up to 70% loss of GβL (Fig. 7, *A* and *B*). Consistent with previous reports (54, 55), both mTORC1 (pS6K^T389^ and pS6^S235/236^) and mTORC2 signaling (pAkt^S473^) were diminished in GβLΔ cells in all three conditions: full media, AA starved (Krebs), or AA starved and leucine-stimulated conditions compared to control cells (Fig. 7, *A* and *B*). We also found that pmTOR^S2448^, but not pERK^T202/Y204^, was diminished in GβLΔ compared with control cells (Fig. 7, *A* and *B*). Thus, we successfully generated GβLΔ cells defective in mTOR activity. To test the role of GβL SUMOylation in regulating mTOR signaling, we transfected HA-GβL WT and HA-GβL full KR mutant (HA-GβL-KR) into GβLΔ cells, followed by AA starvation (–AA, Krebs) and stimulation with 3 mM leucine (+Leu). We investigated mTOR signaling using confocal microscopy/immunofluorescence by measuring intensity levels of pS6^S235/236^ (mTORC1) or pAkt Ser^473^ (mTORC2) in individual GβLΔ cells expressing HA-GβL WT or HA-GβL-KR. In GβLΔ cells expressing HA-GβL WT, we observed enhanced staining of pS6^S235/236^ following leucine stimulation (Fig. 7, *C* and *D*), whereas HA-GβL-KR had negligible pS6^S235/236^ signal (Fig. 7, *C* and *D*). Similarly, GβLΔ cells transfected with HA-GβL WT also showed enhanced pAkt^S473^ compared with HA-GβL-KR-transfected cells (Fig. 7, *E* and *F*). We found no significant changes in mTOR staining between HA-GβL WT or HA-GβL-KR-expressing cells (Fig. 7, *G* and *H*). These results suggest that SUMOylation of GβL has an essential role in controlling mTORC1 and mTORC2 signaling.

## Discussion

In this report, for the first time, our findings demonstrate that SUMO acts as a novel regulator of mTOR activity and complex formation. We demonstrate that GβL, the regulatory subunit of mTOR, is modified by SUMO1, SUMO2, and SUMO3. Our MS analysis identified novel and multiple SUMOylation sites of GβL, which may act as a scaffolding interface in the regulation of mTOR signaling. Consistent with this notion, the putative SUMO sites of GβL are surface exposed in the cryo-EM structure (41), thus potentially modulating protein–protein interactions of mTOR components *via* the SUMO moiety.

Our data indicate that SUMO predominantly mediates nutrient-induced mTOR signaling. While we did not observe a robust defect in mTORC1 activity when *Sumo1*^*−/−*^ MEFs or SUMO1/2/3Δ cells were cultured in nutrient-rich media, we found a strong deficit in mTORC1 and mTORC2 signaling and mTOR complex formation upon leucine stimulation in SUMO1/2/3Δ cells. Thus, SUMO may be necessary to facilitate mTOR component assembly or activation selectively depending on the availability of nutrients.

The mTOR signaling pathway is also influenced by glucose and glutamines (56, 57). The impact of SUMO signaling on mTOR signaling caused by these nutrients is yet to be identified.

Nutrients, such as AAs, induce the rapid localization of Raptor-bound mTOR on lysosomes (58), and it has been shown that nutrients affect mTOR–Raptor interactions only in the presence of GβL (54). Thus, we predict that SUMOylation of GβL may further facilitate this interaction. We did not find that mTOR–GβL interactions are affected in SUMO1/2/3Δ cells, which is not surprising as GβL is constitutively bound to mTOR (54). Our GβL reconstitution experiments, however, implies that the SUMOylation of GβL is necessary to activate mTORC1 and mTORC2 signaling. The mTOR complex components including mTOR, Raptor, and Rictor have conserved SUMO lysine residues. However, the specific timing and impact of these proteins that are affected by SUMOylation and how they will coordinate with SUMOylation of GβL are still unknown.

**Figure 8:**
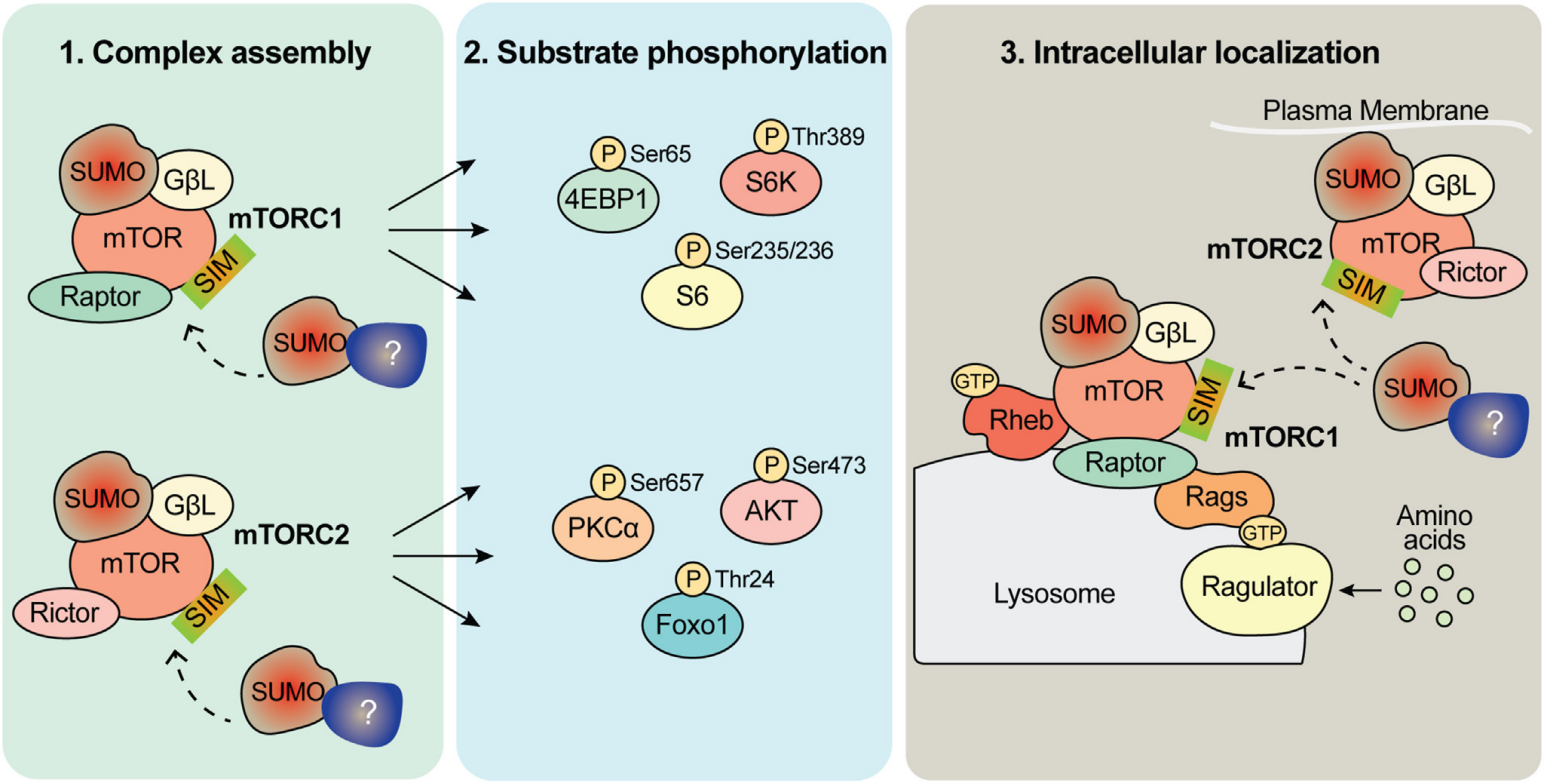
**Schematic depiction of role of SUMOylation in the regulation of mTORC1/2 signaling.** Our model predicts that there are three possible ways that SUMO modification may influence mTORC1 and mTORC2 signaling. (1) mTORC assembly, (2) mTOR substrate phosphorylation, and (3) intracellular localization of mTORC. In addition to GβL, it is possible that one or more additional components of the mTOR and other non-mTOR complex regulators (depicted as question mark [?]) can be modified by SUMO and influence complex assembly and activation. mTOR, mechanistic target of rapamycin; mTORC, mTOR complex; SIM, SUMO-interacting motif; SUMO, small ubiquitin–like modifier.

Mechanistically, we predict that SUMOylation may regulate the interaction of mTOR with its other regulatory components. Interestingly, most SUMO targets including GβL as reported here show low stoichiometry of SUMOylation, which is sufficient to exert cellular and biological functions, although mechanisms are remains not fully understood (17, 59). Thus, we propose that SUMOylation may affect at least three different processes: (1) promote complex assembly of mTORC1 and mTORC2, (2) enhance mTOR kinase activity by promoting substrate recruitment, and (3) regulate proper intracellular localization of mTOR and its components (Fig. 8).

Recent work indicates that K63-polyubiquitination of GβL regulates the formation of mTORC2, where loss of critical ubiquitin modification sites on GβL (K305, K313) promoted the association of mSin1 with mTOR and enhanced mTORC2 assembly and activity (10). While it is possible that SUMOylation *via* unknown protein(s) may similarly mediate both mTORC1 and mTORC2 formation and activity, the mechanistic role of SUMO moiety of GβL in orchestrating the complex assembly and the activity remains to be determined (Fig. 8). Indeed, it is also conceivable that GβL SUMOylation works in coordination with ubiquitination. SUMOylation of GβL may compete with ubiquitination to protect mTOR complex assembly by positively regulating the GβL stability. Such competitive mechanisms are reported in the regulation of the stability of delta-lactoferrin transcription factor (60).

Furthermore, there could be unknown SUMO-modified proteins that may facilitate mTORC formation and activity through SUMO-interaction motifs (Fig. 8). Accordingly, the mTOR kinase has consensus SUMO sites (K425, K873, and K2489) and potential SUMO-interaction motifs (http://sumosp.biocuckoo.org/showResult.php), which might play a role in mTOR activity. We were unable to detect SUMOylation of mTOR in our biochemical methods as SUMOylated mTOR may be present at a low stoichiometry. Furthermore, it is possible that mTOR SUMOylation can be enhanced in the presence of the SUMO E3ligase Rhes, which directly binds and promotes the kinase activity of mTORC1 (15, 35). In addition, SUMOylation of other accessory mTOR components may also influence mTOR signaling, but these prospects warrant further investigation.

Previously, we found that mHTT mediated nutrient-induced mTORC1 activity (36). Since mHTT is SUMOylated at multiple sites (34, 61) and enhances perinuclear association of mTOR in Huntington disease cells (36), we speculate that SUMOylation of mHTT may further facilitate mTOR signaling and affect disease progression in cooperation with SUMOylation of GβL in HD conditions. Likewise, SUMO may induce a multicomplex protein assembly that initiates and sustains nutrient-induced mTOR signaling to orchestrate human diseases, such as cancer, neurological disorders, and neurodegenerative diseases (2, 62, 63, 64). Thus, our study reveals a novel link between SUMO and the mTOR pathway that may impact a variety of biological and disease-associated signaling processes.

## Experimental procedures

### Reagents and antibodies

All reagents were purchased from Sigma unless indicated otherwise. Antibodies against GβL (3274), mTOR (2972, 2983), pmTOR S2448 (5536), pmTOR S2481 (2974), pS6K T389 (9234), pS6 S235/236 (4858), p4EBP1 T37/46 (2855) p4EBP1 S65 (9451), pAkt S473 (4060), p44/42 ERK1/2 (9101), S6K (9202), S6 (2217), 4EBP1 (9644), Akt (4691), ERK1/2 (4695), Raptor (2280), Rictor (9476), SUMO-1 (4930), and SUMO-2/3 (4971) were obtained from Cell Signaling Technology. 6×-His tag antibody (MA1-21315) was from ThermoFisher Scientific. Antibodies for actin (sc-47778), Myc (sc-40), and GST (sc-138 horseradish perxidase) were obtained from Santa Cruz Biotechnology. FLAG antibody (F7425) was obtained from Sigma–Aldrich. HA-tagged monoclonal antibody (901513) was from BioLegend (previously Covance; catalog no.: MMS-101R). HA-tag polyclonal antibody (631207) was from Clontech.

### Cell lines, growth conditions, transfections, and cell proliferation assay

HEK293 cells and MEFs were grown in Dulbecco’s modified Eagle’s medium (DMEM) (11965-092; ThermoFisher Scientific) supplemented with 10% fetal bovine serum (FBS), 1% penicillin/streptomycin, and 5 mM glutamine. For transfections, cells were seeded in 6-well plates or 10 cm plates. After 24 h, transfections were performed with corresponding DNA constructs and PolyFect (Qiagen) using the manufacturer’s instructions. Cells were harvested for experiments 40 h after being transfected. Mouse STHdh^Q7/Q7^ striatal neuronal cells were obtained from the Coriell Institute and cultured in DMEM high glucose (10566-016; ThermoFisher Scientific), 10% FBS, 5% CO_2_, at 33 °C as described in our previous works (34, 36, 46, 65, 66, 67, 68). GβL (sc-425272) and SUMO1/2/3 deletions were carried out in striatal cells using CRISPR–Cas-9 tools obtained from Santa Cruz, as described before (46, 66). Cell proliferation was tested by cell counting kit-8 assay (no. K1018; ApexBio) as per the manufacturer’s instructions. Briefly, control and SUMO1/2/3Δ striatal neuronal cells were seeded in 96-well plate at a concentration 0f 5000 cells per well. Next day, 10 μl cell counting kit-8 solution was added to each well of the plate and incubated at 33 °C for 2 h, and thereafter, the absorbance was measured at 450 nm. The pH of the cell culture media for MEFs, or striatal neuronal cells, remained within the range of 7.4 to 7.6.

### Plasmids and site-directed mutagenesis

pRK5-HA-GβL was obtained from Addgene (1865). His-SUMO1, His-SUMO2, His-SUMO3, and His-SUMO-AA were obtained from Michael Matunis (34). Site-directed mutagenesis was performed on pRK5-HA-GβL using the Quik-Change II Site Directed mutagenesis kit (200523; Agilent Technologies) as per the manufacturer’s instructions. Successful mutagenesis was confirmed by Sanger sequencing (Genewiz).

### Ni–NTA denaturing pull down

Ni–NTA pull down of His-SUMO conjugates was performed as previously described (69). Briefly, cells were rinsed in PBS, scraped from 10 cm dishes, and centrifuged at 750*g* for 5 min. Cell pellets were then directly lysed in pull-down buffer (6 M guanidine hydrochloride, 10 mM Tris, 100 mM sodium phosphate, 40 mM imidazole, 5 mM β-mercaptoethanol, pH 8.0) and sonicated. Lysates were then clarified by centrifugation at 3000*g* for 15 min. All subsequent wash steps were performed with 10 resin volumes of buffer followed by centrifugation at 800*g* for 2 min. Ni–NTA Agarose beads (30210; Qiagen) were pre-equilibrated by washing three times with pull-down buffer. After equilibration, beads were resuspended in pull-down buffer as a 50% slurry of beads to buffer. After quantification of cell lysates, 1 mg of lysate was added to 40 μl of Ni–NTA bead slurry to a total volume of 1 ml in Eppendorf tubes. Beads were then incubated overnight at 4 °C mixing end over end. The following day, beads were centrifuged and underwent washing once in pull-down buffer, once in pH 8.0 urea buffer (8 M urea, 10 mM Tris, 100 mM sodium phosphate, 0.1% Triton X-100, 20 mM imidazole, 5 mM β-mercaptoethanol, pH 8.0), and three additional times in pH 6.3 urea buffer (8 M urea, 10 mM Tris, 100 mM sodium phosphate, 0.1% Triton X-100, 20 mM imidazole, 5 mM β-mercaptoethanol, pH 6.3). Elution was performed using 20 μl of elution buffer (pH 8.0 urea uffer containing 200 mM imidazole, 4× NuPAGE lithium dodecyl sulfate (LDS) loading dye, 720 mM β-mercaptoethanol). Samples were then heated at 95 °C for 5 min and directly used for Western blotting. Inputs were loaded as 1% of the total cell lysate.

### MS

Identification of SUMO modification lysine sites was carried out as described before (42, 43, 44). Briefly, HEK293 cells were transfected with mSUMO3 and HA-GβL, followed by denaturating Ni–NTA pulldown as described previously. The Ni–NTA resin was extensively washed with 50 mM ammonium bicarbonate to remove traces of Triton, and the proteins were digested with trypsin directly on the Ni–NTA solid support for 16 h at 37 °C. The mSUMO3-modified peptides were immunoprecipitated with a custom anti-NQTGG antibody that recognizes the tryptic remnant created on the SUMO-modified lysine side chain, as described before (42, 43, 44). Samples were analyzed on the Q-Exactive HF instrument (ThermoFisher Scientific), and raw files were processed using MaxQuant and Perseus, as described previously (42, 43, 44).

### *In vitro* SUMOylation

SUMOylation assays were performed as described previously (34) in 20 μl using 1× reaction buffer (20 mM Hepes, 2 mM magnesium acetate, 110 mM KCl, pH 7.4), 1 μg of E1 (Aos1/Uba2), 500 ng of Ubc9, 2 μg of SUMO-1/2, 5 mM ATP, 0.2 mM DTT, and 200 ng of Rhes at 32 °C for 30 min unless noted otherwise. GST-RanGAP1 was used as positive control for SUMO E3 ligase activity of Rhes, as described before (15, 34). To stop reactions, 4× NuPAGE LDS sample buffer was added, and samples were heated at 95 °C for 5 min, followed by separation using SDS-PAGE and immunoblotting.

### Purification of His-GβL and GST-GβL

pCMV-His-GβL was cloned from pRK5-HA-GβL into pGEX-6P2 and was transformed into BL21 (DE3) cells (New England Biolabs) and purified using Ni–NTA Agarose beads (30210; Qiagen) according to the manufacturer’s specifications. Briefly, following IPTG induction at 16 °C overnight, BL21 lysate was resuspended in lysis buffer (300 mM NaCl, 50 mM sodium phosphate [pH 8.0], 3% glycerol, 1% Triton X-100, 15 mM imidazole, and 1× protease inhibitor [Roche, Sigma]) and sonicated. Lysate was clarified by spinning at 30,000*g* for 30 min. Ni–NTA beads were pre-equilibrated by washing three times in 300 mM NaCl and 50 mM sodium phosphate (pH 8.0). Beads were incubated with lysate mixing end over end at 4 °C overnight. The following day, the beads were washed three times in an equilibration buffer, followed by elution using 300 mM NaCl, 50 mM sodium phosphate (pH 8.0), and 1 M imidazole.

pGEX6p2-GST-GβL was cloned from pRK5-HA-GβL into pGEX6p2-GST and was transformed into BL21 (DE3) cells and purified using Glutathione Sepharose beads (45000139; Fisher) as described in our previous studies (34, 35, 68, 70).

### Preparation of MEFs

*Sumo1* KO mice (*Sumo1*^*−/−*^) were obtained from Jorma Palvimo (29). MEFs were prepared on E13.5 as described previously (71). Genotyping was performed by PCR with primer pairs for the WT allele (SUMO1 forward: 5′-CTC AAA CAA CAG ACC TGA TTG C-3′; SUMO1 reverse: 5′-CAC TAT GGA TAA GAC CTG TGA ATT-3′) and for the KO allele (Neo1 forward: 5′-CCA CCA AAG AAC GGA GCC GGT T-3′; SUMO-1 reverse: 5′-CAC TAT GGA TAA GAC CTG TGA ATT-3′). The WT of amplicon generated a fragment of 475 bp, whereas the KO amplicon generated a fragment of 550 bp. WT mice (C57BL/6) were obtained from Jackson Laboratory and maintained in our animal facility according to the Institutional Animal Care and Use Committee at The Scripps Research Institute. Mice were euthanized by cervical dislocation, and striatal tissues were dissected and rapidly frozen in liquid nitrogen.

### AA treatments

MEFs were seeded in 6-well plates at least 24 h prior. For essential AA starvation, DMEM were replaced with DMEM/F12 lacking l-leucine, l-lysine, l-methionine, and FBS (F12; D9785, Sigma). After 1 h of starvation, cells were lysed or stimulated with 3 mM l-leucine for 15 min (+Leu). AA treatment in Krebs buffer was carried out as described in our previous work (36). Briefly, striatal cells were placed in Krebs buffer medium (20 mM Hepes [pH 7.4], glucose [4.5 g/l], 118 mM NaCl, 4.6 mM KCl, 1 mM MgCl_2_, 12 mM NaHCO_3_, 0.5 mM CaCl_2_, and 0.2% [w/v] bovine serum albumin) devoid of serum and AAs for 1 h to induce full starvation conditions. For the stimulation conditions, after starvation, cells were stimulated for 15 min with 3 mM l-leucine.

### Western blotting

MEFs were rinsed briefly in PBS and directly lysed in lysis buffer (40 mM Tris [pH 7.5], 120 mM NaCl, 1 mM EDTA, 0.3% CHAPS, 1× protease inhibitor cocktail [Roche, Sigma], and 1× phosphatase inhibitor [PhosSTOP; Roche, Sigma]). Lysates were passed several times through a 26-gauge needle and clarified by centrifugation at 11,000*g* for 20 min at 4 °C. Striatal neuronal cells were washed in PBS and lysed in lysis buffer (50 mM Tris–HCl [pH 7.4], 150 mM NaCl, 1% CHAPS, 1× protease inhibitor cocktail, and 1× phosphatase inhibitor [PhosSTOP]), sonicated for 3 × 5 s at 20% amplitude, and cleared by centrifugation for 10 min at 11,000*g* at 4 °C. Protein concentration was determined with a bicinchoninic acid protein assay reagent (Pierce). Equal amounts of protein (20–50 μg) were loaded and separated by electrophoresis in 4% to 12% Bis–Tris Gel (ThermoFisher Scientific), transferred to polyvinylidene difluoride membranes, and probed with the indicated primary antibodies. Horseradish perxidase–conjugated secondary antibodies (Jackson ImmunoResearch, Inc) were probed to detect bound primary IgG with a chemiluminescence imager (Alpha Innotech) using enhanced chemiluminescence from WesternBright Quantum (Advansta). The band intensities were quantified with ImageJ software (National Institutes of Health). Phosphorylated proteins were then normalized against the total protein levels (normalized to actin).

### IP

For *in vitro* SUMOylation assays containing HA-GβL, HEK293 cells were transfected as described previously and lysed directly in lysis buffer (40 mM Tris [pH 7.5], 120 mM NaCl, 1 mM EDTA, 0.3% CHAPS, 1× complete protease inhibitor cocktail, and 1× PhosSTOP). Lysates were passed several times through a 26-gauge needle and clarified by centrifugation at 11,000*g* for 20 min at 4 °C. IP was performed using Anti-GβL (3274; Cell Signaling Technologies), Protein A/G Plus-Agarose beads (sc-2003; Santa Cruz Biotechnology), and 500 μg of lysate mixing end over end at 4 °C overnight. The following day, beads were washed three times in lysis buffer, followed by a single wash in 1× reaction buffer. Beads were then directly incubated in a reaction buffer containing the aforementioned reaction components for SUMOylation assays.

Striatal cells (2 × 10^6^) were plated in 10 cm dishes, and next day, after leucine stimulation, the cells were washed in cold PBS and lysed in IP buffer (50 mM Tris–HCl [pH 7.4], 150 mM NaCl, 1% CHAPS, 10% glycerol, 1× protease inhibitor cocktail, and 1× PhosSTOP). The lysates were run several times through a 26-gauge needle in IP buffer and incubated on ice for 15 min and centrifuged 11,000*g* for 15 min. Protein estimation in the lysate supernatant was done using a bicinchoninic acid method, a concentration (1 mg/ml) of protein lysates was precleared with 35 μl of protein A/G Plus-agarose beads for 1 h, supernatant was mixing end over end for 1 h at 4 °C in mTOR IgG (2983, Cell Signaling Technology) or control Rabbit IgG (CST-2729S), and then 60 μl protein A/G beads were added and mixing end over end overnight at 4 °C. After 12 h, the beads were washed five times with IP buffer (without protease/phosphatase inhibitor), and the protein samples were eluted with 30 μl of 2× LDS containing +1.5% β-mercaptoethanol and proceeded for Western blotting as described previously.

### Immunostaining

Immunostaining was carried out as described in our previous work (36). Briefly, striatal cells were grown on poly-d-lysine (0.1 mg/ml)–coated glass coverslips, and after 48 h of transfection, the medium was changed to Krebs buffer medium devoid of serum and AAs for 1 h to induce full starvation conditions. For the stimulation conditions, cells were stimulated with 3 mM l-leucine for 15 min. Cells were washed with cold PBS, fixed with 4% paraformaldehyde (20 min), treated with 0.1 M glycine, and permeabilized with 0.1% (v/v) Triton X-100 (5 min). After being incubated with blocking buffer (1% normal donkey serum, 1% [w/v] bovine serum albumin, and 0.1% [v/v] Tween-20 in PBS) for 1 h at room temperature, cells were stained overnight at 4 °C with antibodies against HA (CST, 3724; 1:1000 dilution), pS6^Ser235/236^ (CST, 4858S; 1:200 dilution), pAKT^Ser473^ (CST, 4060S; 1:500 dilution), and mTOR (CST, 2983S; 1:200 dilution). Alexa Fluor 488 (Invitrogen, A21202; 1:1000 dilution) or Alexa Fluor 568 (Invitrogen, A10037; 1:1000 dilution)–conjugated secondary antibodies were incubated together with the nuclear stain 4′,6-diamidino-2-phenylindole for 1 h at room temperature. Glass coverslips were mounted with Fluoromount-G mounting medium (ThermoFisher Scientific, catalog no.: 0100-01). Images were acquired by using the Zeiss LSM 880 confocal microscope system with 63× objective.

### Statistical analysis

Most experiments were performed in triplicate. Images were quantified using ImageJ (FIJI). Data are presented as mean ± SEM. Statistical analysis was performed using Student’s unpaired two-tailed *t* test or one-way ANOVA followed by Tukey’s multiple comparison test (Prism 7, GraphPad software).

## Data and materials availability

All data are available in the article or supporting information.

## Supporting information

This article contains supporting information.

## Conflict of interest

The authors declare that they have no conflicts of interest with the contents of this article.
