# Supplementary material for: SUMO modifies GβL and mediates mTOR signaling
Source: J Biol Chem. 2024 Feb 21;300(4):105778. doi: 10.1016/j.jbc.2024.105778 (PMC10982569; doi:10.1016/j.jbc.2024.105778)
Supplement: Supplemental Figures S1 and S2 Legend [file mmc2.docx]

**Supplementary Figure. 1.** (A) Western blot showing indicated phosphorylation of, PI3K target (pAktT308), and mTORC2 target (pAkts473), phospho and total substrates in WT (Sumo1+/+) and Sumo1 KO (Sumo1¬–/–) primary MEFs grown in F12 (- AA) or starved and stimulated either with either 3mM L-leucine (+ Leu) or 50o nM insulin or both. (B) Quantification of indicated proteins from A. Error bars represent mean ± SEM, *p < 0.05; **p < 0.01 by Student’s-t test comparing WT and Sumo1 KO cells.

**Supplementary Figure. 2.** (A) Western blot showing indicated proteins including phosphorylation of PI3K target (pAktT308) and mTORC2 target (pAktS473) in striatal control CRISPR- or SUMO1/2/3-depleted (SUMO1/2/3Δ) cells stimulated with insulin (500 nM) in DMEM serum free medium. (B) Quantification of indicated proteins from A. Error bars represent mean ± SEM. Not significant (n.s) by Student’s-t test comparing control CRISPR and SUMO1/2/3Δ cells.
