# Supplementary material for: SUMO modifies GβL and mediates mTOR signaling
Source: J Biol Chem. 2024 Feb 21;300(4):105778. doi: 10.1016/j.jbc.2024.105778 (PMC10982569; doi:10.1016/j.jbc.2024.105778)
Supplement: Supplemental Figure S1 [file mmc3.pdf]

Supplmentary figure 1

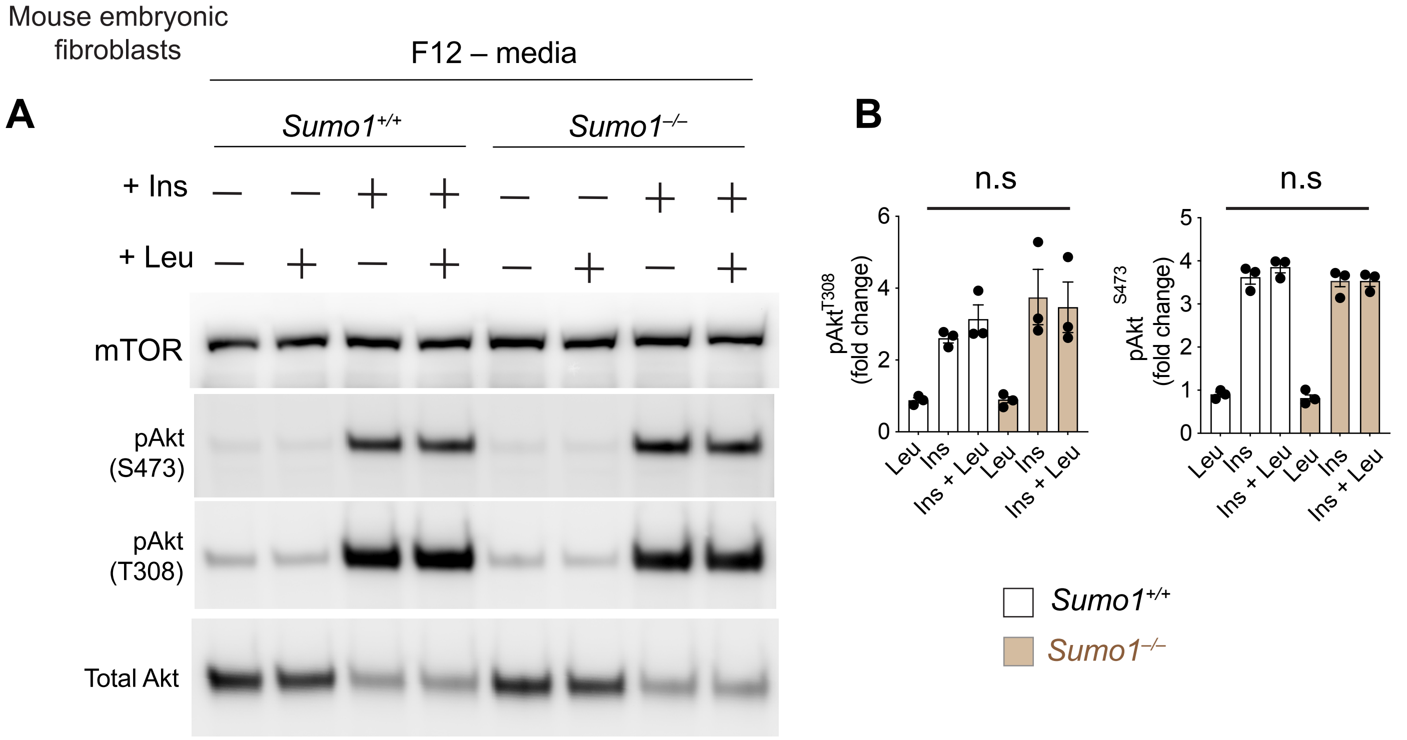

Supplementary Figure. 1. (A) Western blot showing indicated phosphorylation of, PI3K target (pAkt T308), and mTORC2 target (pAkt 473), phospho and total substrates in WT (*Sumo1*$^{+/+}$) and Sumo1 KO (*Sumo1*$^{-/-}$) primary MEFs grown in F12 (– AA) or starved and stimulated either with either 3 mM L-leucine (+ Leu) or 500 nM insulin or both. (B) Quantification of indicated proteins from A. Error bars represent mean ± SEM, not significant (n.s) by Student's-t test comparing WT and Sumo1 KO cells.
